# Clinical assessment of monolithic zirconia crowns fabricated using an intraoral scanner

**DOI:** 10.34172/joddd.41070

**Published:** 2024-09-07

**Authors:** Reza Eftekhar Ashtiani, Leila Nasiry Khanlar, Rahab Ghoveizi, Elaheh Beyabanaki

**Affiliations:** ^1^Prosthodontics Department, School of Dentistry, Shahid Beheshti University of Medical Sciences, Tehran, Iran; ^2^A.T. Still University, Missouri School of Dentistry Oral Health, Kirksville, Mo, US

**Keywords:** CAD-CAM, Dental margin adaptation, Zirconia

## Abstract

**Background.:**

This in vivo study assessed the accuracy of single-tooth monolithic zirconia crowns made using an intraoral scanner (IOS) and computer-aided design/computer-aided manufacturing (CAD/CAM) technology.

**Methods.:**

Thirty patients requiring single posterior crowns were selected. The teeth were prepared with a deep chamfer finish line with a 1-mm extension subgingivally and a 1-mm reduction in all surfaces by one prosthodontist. The gingival margins were retracted using a gingival retraction paste before making impressions using a Trios scanner. All the digital impression files were sent to one laboratory using the dental designer software (3Shape, Copenhagen, Denmark). After completing the milling and sintering processes, the crowns were dyed and glazed. After removing the temporary crown, the qualitative assessment of proximal contacts of definitive monolithic zirconia crowns was performed according to the CDA criteria. Data were analyzed with Friedman’s two-way analysis and independent t-test at α=0.05.

**Results.:**

The difference in axial and total gaps between premolar and molar teeth was not significant; however, the mean marginal gap of molars was higher than that of the premolars (*P*=0.043). Furthermore, the comparison of the axial, total, and marginal gaps between the upper and lower jaws showed no significant difference (*P*>0.05). The distribution of occlusal and proximal contacts indicated 60% and 66.7% proper contacts and no contacts in 6.7% and 10% of cases, respectively.

**Conclusion.:**

Using IOSs could result in accurate monolithic zirconia crowns in terms of adaptation. Also, most occlusal and proximal contacts did not need any adjustments.

## Introduction

 Computer-aided design/computer-aided manufacturing (CAD/CAM) techniques were introduced in the early 1980s to facilitate the process of indirect restoration fabrication, improve the quality of restorations, and also enable the use of esthetic ceramic materials.^1,2^ Use of digital systems requires digitization of oral environment with the help of intraoral or laboratory scanners, software programs to design the prostheses, and manufacturing systems to convert the digital models to the analog structures.^3,4^

 In recent years, new systems for direct intraoral scanning have been introduced to the dental market to digitize the entire workflow.^3^ Some currently used intraoral scanners (IOSs) include iTero, Cerec, Trios, Lava C.O.S, Plan scan, and E4D. These systems are different in critical factors such as principles of work, light source, the necessity of using powder, function, and the output file format.^4,5^ It has been claimed that IOSs are more advantageous for dentists than conventional impression techniques.^6^ IOSs do not require an impression material or tray, which could increase patient comfort^7^ and decrease technical sensitivity. Moreover, the laboratory model fabrication steps are eliminated. Inaccuracies in the impressions can hardly be corrected in the laboratory process, and they negatively affect the internal and marginal fit. Therefore, IOSs potentially increase the accuracy of the final restoration.^8,9^

 The introduction of CAD/CAM systems has also enabled extensive use of zirconia.^10^ Zirconia, a crystallized dental ceramic, has many advantages, including high strength and optimal biocompatibility. On the other hand, due to the white and opaque color of zirconia, it is used as a substructure in crowns fabricated by CAD/CAM systems.^10,11^ The use of the zirconia substructure has problems, such as the need for porcelain coating and a prosthetic technician to apply it, decreasing the standardization of the treatment, increasing the costs, raising the possibility of porcelain fracture, and reducing the survival rate to 92.7% during three years.^12^ To overcome these problems, monolithic zirconia with high translucency and without layered porcelain was introduced to the dental market.^11^ One requirement of a crown material is to cause no or minimal wear of the opposing teeth. According to several studies, when a full-contour monolithic zirconia crown is used, the wear of the opposing teeth would be lower compared to using veneered zirconia crowns.^13-15^

 However, the quality control of the final restoration is the most important factor determining the acceptability of a restoration. Some clinical guidelines are available for this purpose, and CDA guidelines are among the most accredited measures in this respect.^16-20^ Using the CDA criteria, clinicians can determine the acceptability of a restoration based on the clinical assessment of the marginal adaptation, internal fit, anatomical form, and restoration color. This study aimed to clinically assess internal fit and proximal and occlusal contacts of full-contour monolithic zirconia crowns fabricated using 3Shape IOS and digital workflow using the CDA criteria. The null hypothesis was that crowns fabricated by this method are clinically acceptable based on the CDA criteria.

## Methods

 Thirty patients, irrespective of their gender, requiring single crowns for maxillary first premolar and first molar teeth were selected in this in vivo study (15 first premolars and 15 first molars). The selected teeth required prosthetic restoration with adequate tooth structure to allow deep chamfer preparation with a maximum 1 mm of extension into the gingival sulcus. The patients were asked to sign a written informed consent form for participation in this study, which was approved by the Research Ethics Committee at the Shahid Beheshti University of Medical Sciences (IRCT2015030921371N1). The teeth were cleaned, and the shade of the crowns was determined using a Vita classical shade guide (Vita Zahnfabrik, Bad Säckingen, Germany). Primary impressions were made, and a diagnostic wax-up was performed on the study casts. After anesthetic injection, the teeth were prepared with a deep chamfer finish line for full-zirconia crowns with 1 mm of reduction in all surfaces by one expert prosthodontist. For this purpose, the silicone index made from the diagnostic wax-up, and a round-end taper, medium-grit diamond bur were used. The gingival margins were retracted using a gingival retraction paste (Astringent, 3M ESPE, St. Paul, MN, USA) before making digital impressions using Trios IOS (3Shape, Copenhagen, Denmark). Temporary crowns were directly fabricated using a self-curing composite resin material (Luxatemp, DMG GMbH, Hamburg, Germany) and cemented using eugenol-free temporary cement (TempoCem NE, DMG GMbH, Hamburg, Germany). All the files of digital impressions were sent to one laboratory, where the full-contour zirconia crowns were designed using the dental designer software (3Shape, Copenhagen, Denmark) using STL files.

 For designing the crowns, the cement space was considered to be 15 µm at the margins and 40 µm in the axial walls and occlusal surface according to the standard 3Shape recommendations. In the proximal surfaces, the distance with the adjacent teeth was set at zero, and in the occlusal surface, a 10-µm distance was considered from the occlusal surface of the opposing teeth according to the standard 3Shape recommendations. The final STL file was sent to the milling machine (CORiTEC450i; imes-icore GmbH, Eiterfeld, Germany). A highly translucent zirconia blank (Crystal Diamond Zirconia, Digital Dental Co, Arizona USA) was used to mill the crowns. They were then colored with zirconia coloring agents by dipping (Color Liquid Prettau® Zirkonzahn, Gais BZ, Italy). Colored crowns were then sintered in a zirconia sintering furnace (sintering furnace, White Peak dental solution, GmbH & Co. KG, Wesel, Germany) according to the manufacturer’s instructions to 1450 ºC. After completion of sintering, the crowns were stained and glazed at 830 ºC.

 In the second appointment, local anesthesia was administered (if required), the temporary crown was removed, and the zirconia crown was placed after cleaning the prepared teeth for the assessment procedure. First, the qualitative assessment of proximal contacts was made according to the CDA criteria, and four groups were designed as follows.

- Group A: Proper proximal contact was assessed using dental floss. No pressure was applied to the adjacent teeth in this group, and the crown was seated on the preparation margin.
- Group B: Strong proximal contact: The crown was seated on the preparation margin, but the patient felt pressure on adjacent teeth, and dental floss hardly passed the proximal contact (with pressure).
- Group C: Excessive proximal contact: The crown margin did not reach the preparation margin in this group.
- Group D: Open proximal contact

The quality of proximal contact was recorded and then adjusted with a zirconia diamond bur (Komet; Rock Hill, SC, USA) if required. After ensuring that the proximal contacts did not interfere with the complete seating of crowns, the internal adaptation of the crowns was examined. For this purpose, the gap between the prepared teeth and the inner surfaces of the crowns was recorded using the replica technique before any alterations were made to the internal surfaces of the crowns. Low-viscosity silicone (Fit Checker Advanced Blue, GC America Inc., Alsip, IL, USA) was applied to the crown, and the crown was seated with moderate finger pressure. After the material setting was complete, the crown was removed from the tooth, and a regular-viscosity silicone impression material (Exafast, GC America Inc., Alsip, IL, USA) was injected into the set low-viscosity material. After polymerization, the complex of the two materials was removed from the crown, which served as an index to assess the thickness of the gap. Two longitudinal and transverse sections were made to determine the thickness of the replica (which was equal to the gap). Gap was measured in nine different points by one operator: four areas in the margins, four areas in the axial walls, and one area in the occlusal surface, under a stereomicroscope (Leitz, GmbH, Germany) at × 75 magnification.

 Finally, the occlusal contact was clinically evaluated with an articulating ribbon ((ARTI-FOL; Bausch, Cologne, Germany), and according to the CDA criteria, three groups were designed.

- Group A: Acceptable occlusal contact not requiring adjustment
- Group B: Excessive occlusal contact
- Group C: No occlusal contact

Excessive occlusal contacts were adjusted with a zirconia diamond bur (Komet, USA) to achieve maximum intercuspation without any interferences in eccentric movements, if necessary.

 If crowns needed further laboratory modifications, they were returned to the laboratory. Crowns of group D of proximal contact and group C of occlusal contact were excluded from the study, and the digital impression was made again. After achieving acceptable crown requirements, they were cemented with eugenol-free temporary cement (TempoCem NE, DMG GmbH, Hamburg, Germany). The patients were recalled two weeks later, and after ensuring the healthy status of the gingiva and complete adaptation of the crown by taking bitewing radiographs, the crowns were permanently cemented. The crown was first sandblasted with a standard pressure (Cojet, 3M ESPE, St. Paul, MN, USA) to eliminate temporary cement residues for final cementation. The tooth was cleaned with a polishing brush and a non-fluoride pumice paste. An MDP-containing primer (Z Prime-Plus, Bisco, Schaumburg, Illinois, USA) was applied onto the internal surfaces of the crown, and excess material was removed after one minute with an air spray. After isolation, two layers of the bonding agent (All Bond Universal, Bisco Dental Schaumburg, Illinois, USA) were applied on the tooth, allowed 10 seconds, and then, the excess material was removed by air spray. After light polymerization for 10 seconds, the crowns were cemented using a dual-cured luting agent (Duo-Link Universal, Bisco Dental, Schaumburg, Illinois, USA) and light-polymerized for 5 seconds. After removing the excess cement and ensuring complete polymerization, the occlusion and proximal contacts were reevaluated, and if there were no problems, the patient was discharged. The patients were followed up for one year.

 Statistical analyses were performed using SPSS 25 (SPSS Inc., IBM Corporation, NY, USA). The distribution of data was analyzed using Kolmogorov-Smirnov test. Friedman’s two-way analysis was used to compare the gaps on different surfaces. An Independent t-test was used to compare different teeth and arches for axial, total, and marginal gaps. The significance level was set at α ≤ 0.05.

## Results

 According to Friedman’s two-way analysis, there was a significant difference between some tested points at *P ≤*0.001 (Table 1). The gap at the occlusal surface showed the highest median value compared to buccal, lingual, proximal 1 and 2, and axial-proximal 1, which all showed lower values with insignificant differences between each other. Axial-buccal, axial-lingual, and axial-proximal 2 also showed no significant difference from other surfaces (*P* > 0.05).

**Table 1:** Descriptive statistics for measured points according to Friedman’s two-way analysis

	**Median**	**95% CL**	**Range**	**Max**	**Min**
Occlusal	24.20^a^	(23.16‒33.65)	60.20	74.00	13.80
Buccal	15.80^b^	(14.15‒20.32)	34.00	40.00	6.00
Axial-buccal	20.10^ab^	(18.36‒24.45)	34.30	44.00	9.70
Lingual	13.55^b^	(12.37‒19.44)	40.40	44.00	3.60
Axial-lingual	17.95^ab^	(16.14‒22.69)	44.20	53.00	8.80
Proximal 1	17.50^b^	(15.2‒24.16)	51.90	58.00	6.10
Axial-proximal 1	16.05^b^	(15.56‒27.66)	68.60	74.00	5.40
Proximal 2	16.85^b^	(15.03‒26.67)	65.20	70.00	4.80
Axial-proximal 2	18.40^ab^	(16.5‒29.91)	88.80	97.00	8.20

Different letters within each column indicate significant differences.

 In a comparison of the axial, total, and marginal gaps between premolar and molar teeth, only the marginal gap significantly differed (*P* = 0.043), and the mean marginal gap of molars was higher than the premolars (14.82 µm and 20.82 µm, respectively) (Table 2). However, the comparison of the axial, total, and marginal gaps between the upper and lower jaws revealed no significant differences (*P* > 0.05) (Table 3).

**Table 2:** Mean and SD of axial, total and marginal gaps and comparison between molar and premolar teeth according to independent t-test

	**Tooth**				***t***	***P*** ** value**
	**Premolar**		**Molar**			
	**Mean**	**SD**	**Mean**	**SD**		
Axial gap	20.15	5.38	22.02	8.32	-0.706	0.486 NS
Total gap	18.61	3.87	22.10	8.27	-1.404	0.171 NS
Marginal gap	14.82	4.47	20.82	9.39	-2.123	0.043*

* = Significant, NS = Nonsignificant

**Table 3:** Mean and SD of axial, total, and marginal gaps and comparisons between maxillary and mandibular teeth according to independent t-test

	**Jaw**				***t***	***P*** ** value**
	**Upper**		**Lower**			
	**Mean**	**SD**	**Mean**	**SD**		
Axial gap	20.99	6.90	21.54	7.79	-0.205	0.839 NS
Total gap	19.64	6.46	22.01	7.47	-0.927	0.362 NS
Marginal gap	16.82	8.08	20.33	8.08	-1.165	0.254 NS

NS = Non-significant.

**Figure 1:**
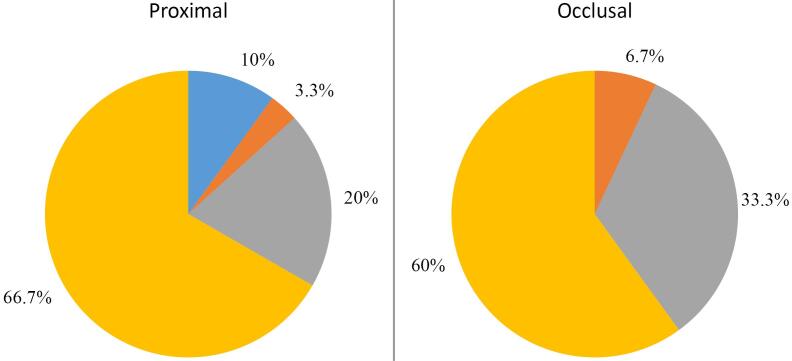
Pie chart showing proximal and occlusal contact scores in all the teeth

 The distribution of occlusal and proximal contacts was shown as pie charts (Figure 1), which indicated 60% and 66.7% proper contacts and 6.7% and 10% no contacts, respectively.

## Discussion

 Monolithic zirconia crowns were used in this study, with no risk of porcelain chipping. Also, the wear of the opposing teeth is lower compared to the porcelain fused-to-metal crowns.^13-15^ Furthermore, zirconia crowns have suitable biocompatibility,^21^ and plaque accumulation on its surface is minimal^22^; thus, adverse effects on the adjacent soft tissue would be minimized. Zirconia does not chemically or physically bond to resin or water-based cements; therefore, the preparation design significantly affects the crown’s retention.^23^ Our findings showed that monolithic zirconia crowns fabricated based on digital workflow according to the CDA criteria had acceptable quality. According to Mclean and von Fraunhofer,^24^ the marginal fit of crowns fabricated in this study was within the clinically acceptable range ( < 120 µm). The median amount of gap was less than 17.5 µm at the margins, less than 20.10 µm in the axial walls, and 24.20 µm in the occlusal surface. Most previous studies reported that the marginal gap observed in crowns made using digital workflow was within the clinically acceptable range.^4,25-30^ Although several factors such as the type of crown, the type of scanner, the amount of gap designed in the software, the design and presence/absence of additional ceramic layering can affect the final fit of the restorations.^31^

 The amount of gap in the present study was lower than that in similar previous studies, which might be attributed to the frequent calibration of IOS and milling machine and the use of a new bur for every 10 crowns. Furthermore, other important factors such as the designed gap (cement space) in the software, tooth preparation design in terms of taper and length, type of material used, and gap assessment method play a role in this respect. However, our findings are not consistent with Dahl and colleagues’ study,^32^ in which the conventional casting method provided better adaptation than the other methods. The difference between their results and ours might be attributed to their small sample size and the gap assessment method, which was based on the triple-scan method.

 The relatively high maximum marginal gap in the proximal area (97 µm) in this study might be the result of a more difficult isolation process in this area, and also the “shadowing effects” in narrow and deep areas, which necessitates multiple and slow scans from different angles, as well as more maneuver of IOS tips to capture the correct forms of the preparation.^25^ Similarly, Ng et al^26^ assessed the IOS impression technique, reporting that the accuracy of this method was lower in proximal areas. They attributed this finding to the presence of saliva and preparation depth in these areas. The supragingival placement of the finish line, if possible, proper isolation, more taper, and disuse of retentive grooves in these areas might be helpful for better capturing of the preparation by the IOSs.

 In the present study, the highest amount of gap was noted in the occlusal surface, which has also been reported in similar studies.^33-35^ This might be due to the amount of gap designed on the occlusal surface (defined by the system) and the incomplete seating of the crown due to friction at the axial walls. An increased amount of gap, especially on the occlusal surface, results in increased cement thickness and subsequently increased risk of de-cementation of the crown.^33-35^ In addition, the marginal gap of the premolar crowns showed less marginal gap compared to the molar crowns. However, there was no difference between the upper and lower jaws in this regard. Since the amount of reduction and the position of the finish line were the same in all teeth and all the axial walls, this finding might be due to the more posterior position of the molars that could minimally limit the use of the IOSs.

 The occlusal surface of 60% of crowns did not require any occlusal adjustment, approximately 33% required minimal adjustment and did not need to be sent back to the laboratory, and around 7% of the crowns required extensive occlusal adjustment and needed to be sent back to the laboratory. Also, around 67% of the proximal contacts of crowns did not require any adjustment, 20% showed strong contacts with minimal adjustment required, about 3% needed excessive adjustment and return to the laboratory, and 10% of the proximal contacts were open. These findings are probably due to the elimination of conventional impression taking, bite registration, and subsequent elimination of common errors related to the tray, the impression material, and the laboratory steps.^25^ Errors in the laboratory steps are related to the processes of pouring the casts, making removable dies, and the sequence of making crowns. Our results were consistent with previous in vivo and in vitro studies.^4,26-30^ Tamim et al^30^ assessed the quality of metal-ceramic single crowns made using iTero IOS impressions using CDA criteria in an in vivo study. They reported that this impression technique yielded clinically acceptable results such that only 20% of crowns required occlusal adjustment, and all the crowns had an excellent fit.

## Conclusion

 Within the limitations of this study, using IOSs could result in accurate monolithic zirconia crowns in terms of internal and marginal adaptation. However, molar crowns showed less proximal marginal adaptation compared to premolar crowns. There was no difference in crown accuracy between the maxilla and mandible. Moreover,most occlusal and proximal contacts needed minimal adjustment, if any, and only 10% and about 7% of the errors (respectively) resulted in the remaking of the crowns.

## Competing Interests

 None.

## Ethical Approval

 This study was approved by the Research Ethics Committee at the Shahid Beheshti University of Medical Sciences IR.SBMU.RIDS.REC.1394.62. This study has registered in Iranian registry of Clinical Trials (identifier: IRCT2015030921371N1).
